# Wearable Devices for Physical Activity and Healthcare Monitoring in Elderly People: A Critical Review

**DOI:** 10.3390/geriatrics6020038

**Published:** 2021-04-07

**Authors:** Eduardo Teixeira, Hélder Fonseca, Florêncio Diniz-Sousa, Lucas Veras, Giorjines Boppre, José Oliveira, Diogo Pinto, Alberto Jorge Alves, Ana Barbosa, Romeu Mendes, Inês Marques-Aleixo

**Affiliations:** 1 Research Centre in Physical Activity, Health, and Leisure (CIAFEL), Faculty of Sport, University of Porto, 4200-450 Porto, Portugal; hfonseca@fade.up.pt (H.F.); joseflorenciosousa@gmail.com (F.D.-S.); lucasdsveras@gmail.com (L.V.); giorjines_boppre@hotmail.com (G.B.); joliveira@fade.up.pt (J.O.); ines.aleixo@ulp.pt (I.M.-A.); 2 Faculty of Psychology, Education and Sports, Lusófona University of Porto, 4000-098 Porto, Portugal; 3 Escola Superior Desporto e Lazer, Instituto Politécnico de Viana do Castelo, 4900-347 Viana do Castelo, Portugal; 4 Laboratory for Integrative and Translational Research in Population Health (ITR), 4050-600 Porto, Portugal; ana.barbosa.02@gmail.com (A.B.); romeuduartemendes@gmail.com (R.M.); 5 Research Center in Sports Sciences, Health Sciences and Human Development (CIDESD), University Institute of Maia, 4475-690 Maia, Portugal; diogoop40@gmail.com (D.P.); ajalves@ismai.pt (A.J.A.); 6 EPIUnit—Instituto de Saúde Pública, Universidade do Porto, 4050-091 Porto, Portugal; 7 Northern Region Health Administration, 4000-477 Porto, Portugal

**Keywords:** sensors, technology, exercise, steeps, bone, cardiovascular, diabetes, cognitive function, sleep

## Abstract

The availability of wearable devices (WDs) to collect biometric information and their use during activities of daily living is significantly increasing in the general population. These small electronic devices, which record fitness and health-related outcomes, have been broadly utilized in industries such as medicine, healthcare, and fitness. Since they are simple to use and progressively cheaper, they have also been used for numerous research purposes. However, despite their increasing popularity, most of these WDs do not accurately measure the proclaimed outcomes. In fact, research is equivocal about whether they are valid and reliable methods to specifically evaluate physical activity and health-related outcomes in older adults, since they are mostly designed and produced considering younger subjects’ physical and mental characteristics. Additionally, their constant evolution through continuous upgrades and redesigned versions, suggests the need for constant up-to-date reviews and research. Accordingly, this article aims to scrutinize the state-of-the-art scientific evidence about the usefulness of WDs, specifically on older adults, to monitor physical activity and health-related outcomes. This critical review not only aims to inform older consumers but also aid researchers in study design when selecting physical activity and healthcare monitoring devices for elderly people.

## 1. Introduction

Worldwide, population aged over 64 years is growing quicker than all other age groups. According to the 2019 revision of the world population prospects, elderly people will represent 16% of the world population by 2050 [1]. Assuring quality of life during the aging process will be one of the most significant social challenges of the twenty-first century, and it includes providing a good healthcare and warranting that elders maintain their capacity to independently perform activities of daily living for the longest period possible, i.e., the so-called active aging process [2]. The use of wearable devices (WDs) and technology to evaluate and monitor physical activity and health-related outcomes in order to maintain overall health, preserve motor control, cardiovascular, metabolic and cognitive performance throughout the aging process will certainly be advantageous [3]. 

Particularly, the use of WDs—essentially composed by a sensor that generates an electrical signal, when reacting to a physical phenomenon, coupled to a transducer (an electronic circuit) that analyzes and transmits it—integrated in accessories, garments, or clothes, allow permanent and pervasive evaluation of physical activity and health-related outcomes [4]. Nevertheless, despite WDs’ massive availability and constant evolution, their actual utility to measure these outcomes and extensive use by the elderly population continues to be limited [5]. 

The rising concern to provide quality of life for elderly people, the high prevalence of chronic diseases in this population, and the heterogeneity related to their health conditions make it urgent to investigate and disseminate the use of valid and reliable WDs, which are adequate to assist elders and researchers during active aging. Accordingly, accurate monitoring of physical activity is an important aspect to precisely assess if elders are accumulating the appropriate amount of sedentary behavior and/or physical activity required to meet the evidence-based public health recommendations, which are needed to mitigate health risks and attain significant health-related benefits [6]. Moreover, together with the substantial increase in life expectancy, the elderly population has a high prevalence of chronic diseases, particularly cardiovascular diseases and diabetes [7], which can be mitigated by adopting a healthy lifestyle that includes no smoking, reduced alcohol intake, healthy body composition, good nutrition, and daily moderate to vigorous physical activity [8]. The use of WDs to help control and attain this healthy lifestyle will certainly be beneficial for all. Lastly, despite the aging process per se, genetic predisposition and other non-modifiable factors are strongly related to lower cognitive performance and dementia onset in elders, increasing evidence shows that modifiable risk factors such as physical activity, social interaction, and cognitive tasks or activities can prevent or delay dementia [9]. Since cognitive performance is associated with the ability to perform activities of daily living independently [10], accurate monitoring of tasks that enhance cognitive function could be a key factor in preserving independence, hence preventing or at least postponing the need for institutionalization.

This review aims to analyze the existing scientific evidence regarding the most commonly used WDs for monitoring: (1) physical activity and energy expenditure; (2) the interactions between physical activity and health-related outcomes in both cardiovascular diseases and diabetes; and (3) cognitive performance, specifically in the elderly population. 

## 2. Monitoring Physical Activity and Energy Expenditure—Accelerometer Wearable Devices

Although the technological elements imbedded in accelerometers may vary considerably among different types and models [11], they are the most commonly used WD evaluating body movement accelerations in one to three orthogonal planes (anteroposterior, mediolateral, and vertical) [12]. Based on this information, accelerometers can provide a wide range of physical activity metrics, both to the researcher and the general public consumer, such as steps count, time spent in different physical activity intensities, sedentary behavior, and daily energy expenditure [6]. Despite the unquestionable usefulness of these metrics from a health point of view, there are some concerns about the validity and quality of the information provided by accelerometers, namely for other populations rather than healthy adults in which most of these metrics we validated, such as the elderly [13]. Researchers have shown that WDs have low accuracy to assess the number of steps performed at low velocities such as those below 2 km·h^−1^ [14,15]. Although this may not be a serious issue in adults or healthy elderly with no mobility limitations, the opposite can be quite true for subjects with substantial gait pattern changes prompted by neurological impairments or other orthopedic or musculoskeletal limitations, such as the elderly, who are frequently cane and walker-users [16]. There have been attempts to overcome this limitation in research-level accelerometers, normally worn at the hip, through the redefinition of the algorithm criteria used to identify steps. For instance, Actigraph, the international research-grade accelerometers leader, developed a low-frequency extension filter to be applied in accelerometry data analysis in order to prevent low-intensity movements derived from slow walking from being discarded. Without these changes, these data would be considered noise (an artifact from the environment) [17] and therefore disregarded from the analysis. Unfortunately, the majority of the WDs manufacturers still estimate the physical activity metrics through proprietary procedures that are undisclosed and work as black boxes. This precludes understanding if these devices take into consideration or not the consumers’ specificities in their analyses [13].

The same type of concerns has arisen about the validity of energy expenditure estimates and time spent in different physical activity intensities provided by consumer-grade activity trackers. Several studies that established WDs validity for the elderly population were based on assumptions that could be misleading, as they often incorporate reference criterion (e.g., energy expenditure equations or moderate-to-vigorous physical activity cut-points) derived from research-grade devices developed for adults and not for the elderly [18]. It is essential that validation studies use as reference criterion a proper gold-standard measure for each physical activity metric being tested [19]. In fact, a study developed by Murakami et al. [20] showed that exergy expenditure assessment provided by several of the mainstream WDs was not comparable with values obtained when exergy expenditure was assessed in a metabolic chamber or by doubly labeled water. Likewise, studies aiming to test the validity of WDs to estimate different physical activity intensities should use as reference criterion indirect calorimetry or, at least, cut-points developed from a calibration study that included a similar population and similar study design settings to those included in the validation study, such as wear placement, age, body composition, physical fitness, and general health condition. Even if proper validation studies demonstrate that a certain WD is able to produce accurate estimates, there is always a real risk that data collected in prospective cohorts could be invalidated if manufacturers change their proprietary and undisclosed algorithm used to estimate these parameters. An illustrative example of this real possibility was the change promoted by Fitbit on the criteria used to account for moderate-to-vigorous physical activities [13,21]. To determine the total amount of minutes of moderate-to-vigorous physical activity, their algorithm initially assumed the sum of all minutes above this intensity threshold. This was later changed to considering only the bouts of at least 10 min of continuous moderate-to-vigorous physical activity. These changes in the way that data is calculated from accelerometers and presented to the end-users can have substantial repercussions for final consumers, healthcare professionals, and researchers. For instance, a healthcare professional aiming to determine if a pharmaceutical or rehabilitation intervention can improve the autonomy of his patients and thereby increase time spent in different physical activity intensities might see his work compromised due to unanticipated criteria changes implemented by the WD manufacturers. As a result, physical activity levels can fluctuate not as a true effect of the health intervention but due to algorithm changes. In order to overcome this limitation, manufacturers should use more transparent data collection and analysis strategies, not only disclosing details regarding the calibration procedures on which the algorithms were based on, which includes the conditions of testing, but also by reporting any changes that might have an impact on the WD measured outcomes. Therefore, algorithm updates should be announced with sufficient anticipation in order to alert and explain the potential impact of these changes for the user. Ideally, algorithms should be developed in a more transparent and universal way through the collaboration between companies and researchers from different fields. This would contribute to avoiding the multiplicity of concurrent approaches and the unnecessary noise resulting from that multiplicity of different strategies to estimate physical activity metrics and their associated validation studies [22,23].

### New Perspectives on Accelerometer Use—Monitoring Mechanical Loading

Apart from being largely used to assess health variables specially oriented for cardiometabolic health such as daily energy expenditure, step counts and time spent in different physical activity levels, recently, the research-grade accelerometers have also begun to be explored as a method to measure mechanical loading related with daily life activities [24,25]. The assessment of these mechanical parameters might be especially important for the elderly population, since physical activity-derived mechanical stimuli play an important role in both bone and cartilage health, which are major health concerns in the elderly [26,27].

Some attempts have already been made to associate bone health and physical activity metrics resulting from research-grade accelerometers. A recent study that investigated the association between bone health and accelerometer-assessed physical activity in older adults found that high-intensity impacts were positively associated with bone strength [28]. In addition to high impact mechanical loading, it seems that sedentary behavior may also be an important variable to consider when monitoring bone health. For instance, Chastin et al. [29] showed that sedentary behavior was negatively associated with total femur bone mineral density in women. Interestingly, it was possible to observe that not only total time spent in sedentary behavior but mainly how this time was distributed throughout the day, which is an information that accelerometers are specifically tailored to provide, was the most important variable explaining bone status. Importantly, the authors showed that the deleterious effects of sedentary behavior on bone health could be minimized by frequent non-sedentary breaks. More recently, another study [24] has shown that WDs based on accelerometers are also capable of very accurately predict ground reaction forces outside controlled laboratory conditions. This new application holds promise to monitor the effects that different types of daily physical activities might have on bone health namely by providing daily feedback to users regarding the achievement of the daily minimum mechanical loading necessary to prevent age and menopause-associated bone mass losses. Nevertheless, the association between these metrics and hard bone health outcomes, such as fractures, has yet to be tested in the elder. If, for one hand, WDs can be used to establish the minimum of exercise required to elicit a positive bone health adaptation, they may also be useful to alert subjects regarding overuse injury risk. This might be particularly useful in contexts such as those when elderly patients with knee osteoarthritis are recommended to engage in a physical exercise program to manage overweight or obesity, but at the same time, it is necessary to avoid high impact activities in order to reduce articular discomfort and osteoarthritis progression. In this case, accelerometer-based WDs would be of great interest, as they could offer instantaneous feedback to the end-user regarding the safety and efficacy of the different exercise activities being performed. Unfortunately, all of these potential applications are not yet available in consumer-grade WDs. This highlights the need for continuous improvement in this field of research as well as the need for better interaction between scientific development and technological companies that market these devices.

## 3. Monitoring Cardiac Function and Cardiovascular Health

Cardiovascular disease is the leading cause of death worldwide accounting for almost 18 million deaths annually [30]. Most patients who suffer from cardiovascular disease are older adults [31]. Reductions in premature cardiovascular disease and mortality require effective management of cardiovascular risk factors, which include reductions in smoking and alcohol consumption, control of weight and high blood pressure, and promotion of healthier diets and physical activity [30]. In fact, the importance of adopting active behaviors to reduce cardiovascular disease and mortality risk has been demonstrated in adults and elderly without cardiovascular disease [32,33]. Several studies have also shown that physical activity reduces all-cause and cardiovascular mortality in patients with cardiovascular diseases, including those with hypertensive heart disease, coronary artery disease, peripheral artery disease, and heart failure [34]. Moreover, adherence to active lifestyles has been shown to be extremely hard to maintain in the long term, especially among older adults and patients with cardiovascular disease, in particular those with advanced stages, more comorbid conditions, and lower physical fitness [35]. To overcome these and other barriers to exercise participation, meta-analysis has investigated the benefits of home-based exercise programs in patients with coronary artery disease and found they can be even more effective in the improvement of exercise capacity compared to center-based supervised exercise programs [36]. Although this suggests that home-based exercise can be an attractive alternative to improve cardiac health in older adults with or without cardiovascular disease, these data also imply that effective and continuous monitoring of the exercise dose may be required to optimize their health benefits and maintain them in the long term. In this sense, many researchers and cardiac rehabilitation centers have explored the merits of telerehabilitation, which employs remote monitoring and communication to deliver and monitor exercise sessions conducted at home, and they showed that this approach might have similar efficacy as compared to traditional center-based programs [37]. Traditionally, hospital or center-based rehabilitation programs use strap heart rate monitors and electrocardiogram (ECG) telemetry systems to monitor heart rate and rhythm during exercise sessions, although their complexity and increased costs make them less convenient and applicable in home settings, which has motivated several researchers and commercial companies to develop novel tools, devices, and strategies to improve the remote delivery of exercise sessions [37]. In the context of exercise, this includes novel devices to monitor heart rate, heart rate variability, and blood pressure. 

### 3.1. Heart Rate Monitoring Devices

In general, exercise guidelines recommend the use of heart rate during exercise sessions to prescribe exercise intensity and ensure safety in older adults and patients with noncommunicable diseases [38]. Measurement of resting heart rate or heart recovery after an exercise session may also be useful to determine the mortality risk and the efficacy of the exercise interventions. Elevated resting heart rate and attenuated heart rate recovery have been consistently associated with increased relative risk of cardiovascular events, and all-cause and cardiovascular mortality [39,40]. Moreover, the risk of all-cause mortality seems to increase with increasing resting heart rate in a linear relation [39]. Even though there is mounting evidence from meta-analysis and randomized-controlled trials that exercise training, especially aerobic exercise, is an effective strategy to reduce resting heart rate and improve heart rate recovery, even in older adults and cardiovascular patients [41,42,43], it is unknown if interventions that use continuous heart rate monitoring to guide exercise prescription are more efficacious in improving heart health and mortality risk reduction. 

The advent of commercially available, wrist-worn, heart rate devices based on optical sensors have provided an opportunity to measure non-invasively and continuously heart rate in ecological environments and over prolonged periods of time at a relatively lower cost compared to traditional and laboratory methods [44]. These WDs are based on a method called photoplethysmography (PPG), which is a low-cost technology that uses optical sensors to measure changes in light-emitting diodes (LED) that are transmitted through the skin surface and reflected back from human tissues to detect volumetric changes in blood circulation. These sensors may use different light colors (i.e., red or green), but most consumer heart rate devices use green light sensors, as they use shorter wavelengths and are more resistant to movement artifacts, making them more convenient to measure heart rate during exercise; however, they also have reduced depth of penetration and produce greater inaccuracies in individuals with darker skins and tattoos [44]. 

A number of studies have attempted to examine the validity of heart rate WDs, but conclusions may be hampered as they employed different devices, protocols, and populations, stressing the need for standardized protocols and measures for a more accurate evaluation of the accuracy and value of these devices [45]. Moreover, some studies compared consumer devices with gold-standard, clinical-grade, electrode-based ECG equipment, while others compared their validity to chest-strap heart rate monitors [45]. Research on consumer optical devices has focused mostly on wearables from Apple, Fitbit, Garmin, Polar, Samsung, Xiaomi, and Huawei [44]. Compared to chest strap heart rate monitors, the majority of studies concluded that most, but not all, wrist-worn consumer devices provide accurate heart rate measurements [46], even in older adults [47], although accuracy varies among different brands over different exercise intensities [48]. In addition, a recent meta-analysis reported that mean differences in heart rate estimates are small and non-significant between wrist-worn and electrode-based or chest-strap heart rate devices during sleeping, resting, treadmill, and daily living conditions [49]. Nonetheless, the absolute error was small in resting conditions but increased during treadmill activities [49]. In addition, mean differences were found to be larger and significant during resistance exercise and cycling [49]. Other studies also reported that the accuracy of some wrist-worn devices can be compromised during activities involving less wrist motion such as cycling [50], while others seem to perform well [37]. The behavior of these devices during exercise has produced conflicting results, with some studies suggesting that during higher exercise intensities WDs are less accurate, while others have reported similar accuracies during resting and vigorous physical activity [44]. Given that heart rate measurement with PPG devices may be influenced by motion artifacts, it is possible that changes in exercise intensity may produce more error as a result of increased arm movements during activity. The European Society of Cardiology (ESC) working group on e-Cardiology also concluded that accuracy differs among devices and decreases with increasing levels of exercise and that smartwatches with PPG technology tend to be more sensitive to motion artifacts than ECG and chest-strap heart rate monitors [45]. Moreover, in cardiac patients, wrist-worn devices have been reported to be less accurate than electrode-based and chest-strap heart monitors, leading to the general conclusion that electrode-based equipment should be preferred when precise heart rate measurement is vital to assure patient safety [37].

### 3.2. Heart Rate Variability Monitoring Devices

Heart rate variability (HRV) analysis is a non-invasive technique that measures the variation in the time interval between consecutive heart beats, which is also known as the inter-beat (R–R) interval and provides information about the state of autonomic nervous health. The autonomic nervous system modulates the cardiovascular system to cope with physical and physiological challenges through the activities of the sympathetic and parasympathetic nervous systems [51]. Depressed HRV is a strong and independent predictor of a number of health conditions, including diabetes, metabolic syndrome, and cardiovascular diseases, as well as increased risk of cardiovascular event and all-cause mortality in patients with chronic diseases and healthy populations [52,53]. HRV decreases with aging, and it can also provide important information about the cardiac health of older adults, as abnormalities are also strong predictors of death in this population [54]. In addition, HRV reduction has been found to relate to a wide range of psychiatric disorders, including acute and chronic stress, anxiety, depression, bipolar disorder, and schizophrenia [55,56,57]. The quantification of the autonomic nervous system activity and the contribution of the sympathetic and parasympathetic domains are achieved through the calculation of several time-domain, frequency-domain, and non-linear metrics [58]. Time-domain metrics measure the amount of variability, which is observed in a sequence of inter-beat intervals (IBI), whereas frequency-domains metrics indicate the amount of absolute or relative power across four frequency bands, namely ultra-low frequency (≤0.003 Hz), very low frequency (0.0033–0.04 Hz), low frequency (0.04–0.15 Hz), and high frequency (0.15–0.4 Hz). Non-linear measures give information about the unpredictability of IBI time series [59] The HRV recording period can range from less than a minute to more than 24 h and are generally categorized into ultra-short term (<5 min), short-term (≈5 min), and long-term recording (≥24 h) [60]. The long-term recordings are considered to be the standard for clinical assessment given their validated ability to predict clinical outcomes; however, ultra-short term recordings could be ideal for the ambulatory assessment of HRV under stationary and non-stationary conditions given their shorter time requirements [60]. Nonetheless, the routine use of ultra-short term recording in health and fitness assessment still depends on a greater understanding of their physiological importance for health and fitness, the identification of the metrics that more strongly associate with health and fitness outcomes, the determination of reference values according to gender and age groups, efficient automatic artifact correction algorithms, and determination of the minimum time to estimate ultra-short term metrics [60]. The standard HRV assessment methods generally encompass sophisticated and expensive multi-lead ECG systems, which require trained staff. In contrast, the rise of consumer WDs, which can be used on the wrist, chest, wrist, or finger, provided the opportunity to monitor HRV in a practical, simple, and regular basis and measure the impact of active lifestyles on autonomic health. For instance, one study that collected data from Fitbit consumer wrist-worn devices in more than 8 million users showed that increased daily physical activity across age is associated with improved HRV in a dose-dependent manner [61]. These results are in line with another large longitudinal study that included 985 older adults and showed that higher total leisure-time physical activity, walking distance, and walking pace are associated with better indices of 24-h Holter HRV [62]. HRV measured with wrist-worn devices has also been related to objective and self-reported measures of physical function in older adults [63], suggesting that these devices may also help to capture the status of overall health in this population.

Some WDs use chest straps to measure HRV while others use PPG technology, which derives pulse rate variability (PRV) as a surrogate [64]. Chest-strap devices use sensors to measure the electric activity of the heart and have been often used as a simple and less costly alternative to traditional ECG. A number of studies that used chest-strap heart-rate devices found that in general, they show excellent agreement during resting and exercise at light to moderate intensities, but some decreased their accuracy during higher exercise intensities, while others maintained an excellent performance even during high-intensity exercise [65,66]. Nonetheless, the overall level of agreement varied among participants, level of exercise, and indices of HRV [65]. The reasons and significance behind these conflicting results are not entirely clear.

On the other hand, the PPG technology measures the time of propagation of pulse waves from the heart to the peripheral circulation, which is a measure known as pulse transit time, and it allows the determination of pulse to pulse (P–P) interval, which is a proxy measure of the R–R interval. Most evidence supports PRV as a valid surrogate of HRV in healthy and younger adults at rest [67]. However, research should confirm this relationship in older adults, especially in those with cardiovascular diseases, as aging is associated with increased arterial stiffness and blood pressure, which in turn are inversely associated with pulse transit time [68]. Indeed, even though often used interchangeably, it has been demonstrated that PRV and HRV metrics may differ under some circumstances, such as ambient temperature, respiratory patterns, posture, and exercise, which seem to be related to changes in hemodynamics, blood pressure, vascular tone, and pulse transit time [69]. Moreover, physical and mental stress seems to deteriorate the agreement between PRV and HRV, in particular short-term variables [67]. In this sense, a systematic review concluded that the correlation between PRV derived from consumer wearables and HRV measured from ECG ranged from very good to excellent under resting conditions; however, it declined progressively with increasing level of exercise [64]. Nonetheless, even at the peak exercise, correlation between measures obtained by Holter and WDs was found to be strong [64]. Several reviews have pointed out the difficulties and limitations of assessing HRV during exercise and suggested that especially spectral analysis can provide inaccurate and unreliable results particularly during higher intensities [70]. Other limitations related to wrist-worn devices may also affect their accuracy, including exercise protocol, sampling rate, sensor positioning, motion artifacts, respiratory patterns, skin photosensitivity, melanin concentration and pigmentation, wrist hair, sweating, and body composition [71,72]. Software differences, proprietary algorithms, and data handling may also lead to different results between devices [64]. Therefore, standardization of protocols and measures is clearly needed for a more solid assessment of the accuracy of consumer devices, especially in older adults.

### 3.3. Blood Pressure Monitoring Devices

Despite raised awareness and advances in medical treatment, the prevalence of hypertension is constantly rising due to the aging population, with the world facing a growing population as the number of older adults continues to grow [73]. Ambulatory blood pressure monitoring (ABPM) is a non-invasive automated technique that allows the assessment of blood pressure over extended periods of time (i.e., 24 h) and therefore plays a central role in the diagnosis and management of hypertension [74]. Unlike office blood pressure, ABPM permits the identification of particular blood pressure variation patterns, such as masked and white hypertension as well as nocturnal hypertension, morning blood pressure surge, and those with dipping, reverse dipping, and non-dipping blood pressure [74]. Moreover, there is considerable evidence to suggest that home blood pressure monitoring (HBPM) and ABPM provide greater prognostic information concerning cardiovascular outcomes than office blood pressure, including in older adults [75,76,77]. Given that ABPM involves several readings during the day and evening, many patients report moderate to severe discomfort and restriction of their daily activities with this method [78], decreasing their acceptance and adherence to regular blood pressure monitoring. With the evolution of digital health technology and artificial intelligence in blood pressure assessment, the number of WDs that allow everyday non-obtrusive readings of blood pressure is growing, some of which have been validated according to international clinical standards [79]. Coupled with the additional capacity of measuring other behavior and physiological markers as well as environmental conditions, these devices may add greater context information for the diagnosis and management of high blood pressure. The underlying technology varies among WDs, and it includes oscillometric, applanation tonometry, PPG, ultrasound, and bioimpedance methods [79]. Some of these promising WDs include wrist watches that use oscillometric-based techniques to measure blood pressure, two of which have been validated to be used in the sitting position with the wrist at heart level [80]. The Omron HeartGuide showed acceptable readings of office and out-of-office blood pressure compared to ABPM [81]. Other studies reported that wearable cuff-less devices, which use PPG technology, also show good accuracy and high correlation with manual blood pressure measurements [82], one of which has been validated under static and dynamic conditions and showed high fidelity to rapid changes in blood pressure as well as greater preservation of sleep quality during blood pressure monitoring overnight [83]. Even though evidence is sparse for the validity of WDs to measure blood pressure during exercise, more so in older adults, these devices can still play an important role in capturing the effects of active lifestyles on blood pressure and guide exercise prescription, as it has been recently demonstrated that blood pressure self-monitoring before and after exercise leads to improve exercise adherence among adults with hypertension [84].

## 4. Monitoring Type 2 Diabetes-Related Outcomes

Diabetes mellitus is a global public health problem, constituting one of the most relevant noncommunicable diseases [85]. Type 2 diabetes (T2D) accounts for around 90% of all diabetes types, comprising the most frequent form of this disease, and it has been increasing globally. This rise is driven by increased exposure to unhealthy behaviors, such as physical inactivity and sedentary behavior and the consumption of unhealthy foods. Projections for 2045 reveal an alarming increase in the prevalence of diabetes in all age groups and both genders [86].

Diabetes has a significant economic impact on individuals, families, and health systems due to related direct and indirect costs (e.g., microvascular and macrovascular complications, reduced life expectancy, poorer quality of life, premature death and disability, labor-force dropout, absenteeism and presenteeism), and it is expected to increase by 2045 [87,88,89].

One of the fundamental elements for T2D control is regular physical activity (PA) [86]. Several organizations recommend at least 150 min/week of moderate-intensity aerobic exercise and 2–3 sessions/week of resistance exercise on non-consecutive days [90,91,92]. Community-based exercise programs are widely recommended to increase PA levels in middle-aged and older individuals with T2D [93,94], especially to maintain their long-term adherence to exercise programs. Older individuals with T2D frequently have medical conditions that may predispose them to exercise-related injuries and adverse events, mainly due to poor glycemic control and diabetes-related comorbidities [95]. Therefore, a rigorous assessment of individuals’ medical history and conditions should be performed, and careful monitoring should be provided [96].

### 4.1. Blood Glucose Monitoring Devices

Patients with T2D and healthcare providers should guarantee safety conditions before PA practice [91,96,97]. Usually, patients are encouraged to self-monitor their blood glucose levels through capillary blood glucose, using a glucometer and related supplies (e.g., test strips, lancets and lancing devices, alcohol swabs). However, the repeated daily process can become uncomfortable, therefore discouraging glucose assessment and PA practice [97]. Nevertheless, there are new methods to measure blood glucose levels without repeated capillary punctures—the continuous glucose monitoring systems—which measure glucose levels in the interstitial fluid and allow the management of the acute glycemic control, thus improving glycated hemoglobin [97,98]. 

Currently, there are two main WDs for continuous blood glucose levels: (1) a system consisting of a smartphone app or a handheld reader and a glucose sensor that uses a thin and flexible filament inserted under the skin, on the back of the upper arm [99]; and (2) a system that uses a one-touch applicator that inserts a small sensor beneath the skin and a slim sensor that continuously measures glucose levels and sends data wirelessly to a display device (a small touch screen receiver or compatible smart device) through a transmitter [100,101,102,103]. Both of these devices share similar characteristics: some require daily calibration [100,101,102,103]; sensors last from 7 [100,101] to 14 days [99]; transmitters may be replaced between three months [101] to one year [103]; and they are waterproof up to 2.4 meters for 10-min periods [103]. These devices automatically obtain frequent glucose data that is manually scanned or wirelessly transmitted to a nearby receiver to display the readings (monitor) [97].

Compared with traditional methods, these WDs have the main advantages of automatically assessing glucose levels in 5-min intervals, generating alerts according to the values and making adjustments accordingly, and creating patterns to help in decision-making by individuals and healthcare providers regarding medication, nutrition, and PA [104]. Some of these devices integrate insulin pumps and allow for insulin supply adjustments in response to glucose variations [103]. 

Apart from being more expensive and requiring skills for its use and reading, compared with traditional methods [104,105], continuous glucose monitoring systems do not cause pain, they are not dependent on patient’s compliance to glucose assessment, nor do they demand supplies other than the sensors, which often have associated costs [97].

With all these benefits, continuous blood glucose monitoring systems have the potential to be used in individuals with T2D while practicing exercise, allowing for real-time glucose monitoring and immediate corrections if necessary, hence increasing their safety (pre-, during, and after exercise practice) and contributing for T2D control. 

### 4.2. Foot Temperature and Plantar Pressure’s Monitoring in Type 2 Diabetes

Growing evidence suggests that PA practice can positively impact diabetic foot risk—which is a frequent complication in patients with T2D, namely in the improvement of nerve velocity conduction, peripheral sensory function, foot peak pressure distribution, and ulcer incidence reduction [106].

Nevertheless, for individuals with high-risk of diabetic foot (those with high diabetic peripheral neuropathy, peripheral vascular disease, abnormal plantar pressures, abnormal gait, hyperglycemia, hypertension, and dyslipidemia), and particularly in the elderly, some cautions are needed, i.e., adequate foot care, footwear, and medical follow-up [107]. In the past years, a set of WDs has been designed to assess several risk factors for diabetic foot, namely foot temperature and plantar pressure [108]. 

For foot temperature assessment, new WDs are accessible: home foot temperature sensors [108]; wireless mats that remotely measures the temperature of the plantar surface of the foot with minimal engagement from the individual [109]; infrared thermal imaging cameras linked to smartphones [110]; and insole devices or optical fiber-based smart textiles (smart socks or insoles) [111]. Although the effectiveness of these devices seems promising, more research is still needed [108,112].

Regarding plantar pressure monitoring, appropriate footwear is recommended to decrease plantar pressure, which can be measured by pressure plates or insoles with pressure sensors [108]. Smart insoles are being developed with several pressure sensors, so it is possible to monitor plantar pressures and provide alerts directly to the users through a smartwatch [113]. 

These WDs can be useful in the context of PA monitoring, not only promoting T2D control but also minimizing foot injuries mainly in the elderly.

## 5. Monitoring Cognitive Performance and Brain Function

The implementation of efficacious methods for cognitive assessment is crucial to study possible interventions against the cognitive decline that frequently characterizes aging and even more neurodegeneration. For instance, instruments to screening cognitive impairment in older adults are commonly questionnaires that test for orientation, memory, language, attention, visuospatial, and other components depending on the instrument (for refs see [114]). Although they are cheap and easy tools to apply to large population groups, these self-report tools do not always directly translate real-life human behavior and cognition. 

Cognitive function monitoring in non-laboratory settings has been asserted as an important alternative to conventional cognitive test batteries. Mobile platforms (smartphones and tablets) are being used for cognitive assessment of older adults, improving repeated and continuous assessment [115]. Additionally, cognitive assessment through serious video game playing has been tested in multiple populations, including children and adolescents [116], adults and older adults [117,118] and as possible tools for cognitive screening in neurodegenerative diseases [119]. The potentiality of exergames that promote physical activity with and without combined cognitive training for cognitive decline prevention and neurorehabilitation remain to be further explored [120], particularly in older adults. 

Techniques currently available for brain monitoring, as an increasingly essential concern in the context of physical activity and health, include WDs that can directly measure brain electrical activity and brain function. These wearable headsets allow electroencephalographic (EEG) measurement of brain activity and can provide immediate neurofeedback that can be summarized and presented to the user [121]. Research aiming to explore the pattern of brain activity resulting from pathophysiological aging can take advantage of continuous and real-context EEG mapping and the analysis of EEG patterns under different stimulus. Using a large sample of participants (*n* = 6029, 18–88 years of age), Hashemi et al. [122] suggested an age- and sex-related EEG power and frequency alterations. 

Studies on neuro-electric alterations in healthy older adults are often retrospective, taking into account lifelong physical activity or measuring older adults with sedentary lifestyles versus those who exercise regularly (for refs, see [123]). For instance, Olsen et al. [124] monitored EEG, recorded from 64 scalp sites, while twenty-seven healthy participants exercised on a cycle ergometer at low and moderate intensities (40% and 60% VO_2_ peak). From our knowledge, few studies have conducted an EEG evaluation during physical exercise practice, and none using wearable headsets. In fact, the reliability, accuracy, and precision of EEG trace using WDs must be examined to ensure that general physiological responses to physical exercise, such as increasing heart rate and brain blood vessels pulse (due to the electrode placement), frontal scalp muscle activity, head and body movements and perspiration, do not cause the biological artifacts previously described [125]. 

A growing body of evidences has focused on the ability to measure aspects related to brain function including sleep patterns, gait, and cognition, which are influenced by physical exercise habits. Smartwatches propose frequent cognitive assessment in remote settings through simple cognitive tests [126]. Recent literature suggests that smartwatch-based cognitive tests are able to measure attention, working memory, and executive function in healthy young individuals. Importantly, these tests can be accompanied by smartwatch-based sensor data collection, allowing the simultaneous collection of cognitive, physiological, and behavioral data [127]. From our knowledge, no information regarding the usability, acceptance, and validation of smartwatch-based tool for cognitive assessment in older adults with or without dementia has been published. 

Sleep architecture and continuity are fundamental to brain health, and sleep disorders have been linked with neurodegenerative diseases [128]. Nevertheless, exercise has been suggested as a potential nonpharmacological treatment for insomnia or sleep complaints in older adults (for refs, see [129]). Sleep and wake patterns can be monitored by assessing the basic structural organization of normal sleep cycle; sleep quality and quantity, circadian rhythmicity, sleep consolidation, regularity, and napping are important factors [121]. Depending on the experiment design and aim, different wearable sleep-trackers such as wristbands, armbands, smartwatches, headbands, rings, and sensor clips [130] are available. For instance, sleep biomarkers can be recorded continuously using a commercial headband worn overnight on the forehead with sensors that measure EEG, electrooculography (EOG), surface electromyography (EMG), ECG, pulse rate, head movement, and snoring incidence [128]. The first studies evaluating the performance of wearable sleep trackers against standard polysomnography (PSG) go back to 2012 (for refs see [130,131]). These studies were conducted mainly in children and adults with or without some kind of disorder, and no studies were conducted in older adults. It has been identified the need for independent validation of WDs to support and advance sleep research in general [131] and the better understanding of links between sleep and daytime behaviors such as physical exercise [130], particularly in older people. 

Evidence from clinical studies shows that gait abnormalities have been associated with the risk for the development of dementia [132,133]. As previously mentioned, temporal and spatial gait parameters are often carried out at specialist centers; however, alternative approaches, measuring gait remotely in living settings, might provide additional information on gait performance and enhance the possibility of large-scale gait evaluation [134]. Several promising areas of innovation offer easy-to-use remote systems for objective assessment and estimation of gait endpoints, including cadence, gait speed, double support, lateral step variability, foot strike angle, toe-off angle, stance, step duration, stride length, swing velocity, and toe-out angle. For instance, the incorporation of pressure sensors and accelerometers into footwear insoles can provide a newer approach to gait analysis, although external factors and the context associated with the data collection should be taken into account [121]. 

More research is needed concerning the application of neuroscientific knowledge into non-invasive daily life applications and tools in the context of active aging living and physical activity and exercise. By providing macroscopic measurements of brain activity and function and human behavior, these emerging devices and methods have the potential to not only uncover the pathophysiology features of the aging process but also provide new digital health-related biomarkers for the development and monitoring of physical activity and exercise interventions, and other nonpharmacological trials. 

## 6. Future Research Perspectives

The interaction between the so-called personalized digital medicine [135] and the constant evolution of new types of sports-related WDs [136] sets the stage for a new working paradigm regarding the evaluation of the physical activity and exercise effects on health-related outcomes in elderly people. At the same time, the growth of the Internet-of-Things (IoT)—a network of WDs and other technological devices with interconnected autonomous communication [137]—will certainly create the required interface to a more permanent, objective, and holistic monitoring, enhancing the effectiveness of the different active aging strategies, and helping elders, researchers, and caregivers to effectively intervene. 

Scrutinizing non-invasive strategies of collection and analysis of biomarkers, accurately providing information regarding the acute and chronic physiological effects promoted by physical activity and exercise is mandatory, particularly in the aging population. Despite their own limitations, when compared to invasive methods such as blood collection, many advances have been made when using sweat, tears, urine, and saliva [138,139,140] to precisely monitor physiological alterations and health status in sports and exercise medicine. For instance, important biomarkers that characterize health status and sport-related adaptations (e.g., muscle damage, inflammation, cardiovascular stress, oxidative stress, immune response, and hydration state) are present in both urine and saliva [140]. Moreover, physiological alterations of skin temperature and sweat constituents are highly valuable to understand an individual physiological state and can be used as a diagnosis for common pathological situations and physical activity-related effects, e.g., hyponatremia, hypokalemia, dehydration, glucose monitoring, pressure ischemia (lactate in sweat), and pressure ulcers (temperature) [139]. Despite the limited commercial availability, these skin-worn devices have been rapidly evolving. They are becoming more mechanically resilient and flexible, and they are also capable of measuring different hemodynamic and metabolic parameters simultaneously. A recent study showed that a single skin-worn device was able to accurately measure the physiological effects of a meal ingestion and an exercise session, specifically measuring glucose in interstitial fluid, amount of lactate, caffeine, and alcohol present in sweat, and blood pressure and heart rate changes [141]. Curiously, none of these recent studies have been conducted on elders.

These new WDs will be useful to monitor the effects of daily activities in the elderly, especially those with underlying health conditions such as musculoskeletal and cardiovascular diseases, type 2 diabetes, and cognitive impairment. Moreover, real-time collected data will certainly improve the users’ self-awareness of their health conditions and also be valuable to their caregivers, especially upon anomalous physiological alterations. Consequently, the integration and scientific validation of these different WDs and technologies to specifically monitor elders seem mandatory.

## 7. Challenges and Conclusions

Despite the growing number of studies analyzing WDs utility and their diverse applications in different aspects of the elders’ lifestyle and healthcare monitoring, many limitations and challenges are still present. These limitations not only affect WDs use in this population but also other populations and different diseases. This highlights the need for further research about which WDs and future technologies could be developed to serve the current needs of physicians, and other specialists, to optimize older adults’ healthcare treatment in different settings. 

Elders’ capacity to acknowledge the importance of and properly use WDs also remains a challenge. This acceptance challenge must support the development of easy-to-use and comfortable WDs and IoT technology with simple hardware and software. An adequate balance between WDs usage parameters, performance, and accuracy are mandatory.

Affordable and easy-to-use WDs to monitor lifestyle and health-related characteristics of the ever-expanding elderly population will not only be necessary but eminently needed to overcome the diverse necessities imposed by active aging. This massive social challenge will require that both researchers and different professionals from the diverse healthcare and fitness settings use accurate and valid WDs, and most importantly, that independent elders acknowledge their utility, empowering this population to monitor and manage their lifestyle. 
